# Treatment outcome and factors associated with mortality due to malaria in Munini District Hospital, Rwanda in 2016–2017: Retrospective cross-sectional study

**DOI:** 10.3389/fpubh.2022.898528

**Published:** 2022-08-09

**Authors:** François Hakizayezu, Jared Omolo, Emmanuel Biracyaza, Joseph Ntaganira

**Affiliations:** ^1^Department of Epidemiology and Biostatistics, School of Public Health, University of Rwanda, Kigali, Rwanda; ^2^Centers for Disease Control and Prevention (CDC), Field Epidemiology and Laboratory Training Program (FELTP), University of Rwanda, Kigali, Rwanda; ^3^Programme of Sociotherapy, Prison Fellowship Rwanda, Kigali, Rwanda

**Keywords:** severe malaria, death, epidemiology, risk factors, hospital

## Abstract

**Introduction:**

Malaria is a major public health burden in developing countries despite efforts made by several countries. This disease leads to high morbidity and mortality among Rwandans, particularly in the Southern Province where it was the sixth national cause of morality; at Munini hospital it is the first cause of mortality, but the associated factors remain unknown. In this study, we determined the factors associated with deaths among patients with severe malaria to come up with evidence-based interventions to prevent malaria and its factors.

**Methods:**

A retrospective cross-sectional study was conducted on malaria patients who were treated at the Munini District Hospital from 2016 to 2017. Data were collected from the hospital records or registers relating to patients who were admitted with severe malaria. The odds ratio was estimated by bivariate logistic regression and multivariate hierarchical regression models for determining the associated factors of deaths. Data were analyzed using STATA/MP Version 14.1 and Epi-info with proportions.

**Results:**

The study population were mostly women (*n* = 237, 59.1%), farmers (*n* = 313, 78.05%), aged 16–30 years (*n* = 107, 26.68%). Our results indicated that the majority of deaths were women (56.25%). Socio-economic and clinical determinants are important predictors of death among patients with severe malaria. Patients with coma had higher odds of dying (AOR = 7.31, 95% CI :3.33–16.1, *p* < 0.001) than those who were not. The possibility of mortality increased by almost four times in patients who delayed consultation by a day (AOR = 3.7, 95%CI:1.8–4.1; *p* < 0.001) compared to those who came in very early. Patients who had severe malaria in the dry season were at a lower risk of mortality (AOR = 0.23, 95%CI:0.08–0.64, *p* = 0.005) compared to those with severe malaria during the rainy season.

**Conclusion:**

Lack of health insurance, age of the patient, delayed diagnosis, coma, proximity and access to healthcare services, and weather conditions were the major factors associated with mortality among patients with severe malaria. Comprehensive, long-term, equity-based healthcare interventions and immediate care strategies are recommended.

## Introduction

Malaria is a global public health burden with 250 to 500 million reported cases each year (1), of which half occur in developing countries, where high mortality due to severe malaria is regularly documented. The onset of severe malaria and fatal disease, not totally restricted to Plasmodium falciparum infections, can lead to mortality. The important health complications of malaria that can lead to mortality include celebral malaria, pulmonary edema, acute renal failure, severe anemia, bleeding, acidosis, and hypoglycemia (2, 3). Reports indicate that 216 million cases of malaria occurred worldwide resulting in global mortality of 445,000 in 2016. Among them, 90% were reported in Africa and 90% of all malaria-related deaths occurred in Sub-Saharan Africa (2, 4, 5). Malaria mostly kills children under five. Recent studies estimate that at least 20% of all deaths in children under five in Sub-Saharan Africa (SSA) are due to malaria infection (6). In the malaria-endemic countries of SSA, 30% of all outpatient visits are for malaria, and between 20 and 50% of all hospital admissions are due to complications from malaria (7). Poor people are at a higher risk of getting infected and reinfected more frequently (8). The burden of malaria in Rwanda is characterized by its morbidity rate and mortality rate of 18.3 and 5%, respectively in 2015 (9). In addition to the high rate of mortality due to malaria, patients recovering from severe malaria continue to face several short and long-term health complications that include anemia, coma metabolic acidosis, hypoglycemia, hyperlacticacidemia, seizures, febrile convulsion, and other complications affecting the central nervous system (10–12).

In eastern African countries, different healthcare interventions have been adopted for reducing the high prevalence of malaria and related mortality. Several studies have established that the incidence of malaria is reduced due to the preventive measures taken for combating the burdens of this communicable and negligible disease (11, 13). However, it was recently documented that its prevalence continues to remain high and that its burden leads to increased mortalities (11, 14, 15). Several factors were documented as predisposing malaria patients to poor treatment outcomes. These include impaired consciousness, respiratory pain, season, hypoglycemia, and jaundice (11, 16–19). A review of the literature indicates that the prevalence of malaria has reduced in countries such as Burundi, Rwanda, Kenya, Tanzania, South Sudan, and Uganda but studies indicated that the prevalence of mortality due to malaria remained high in the region. A study in Uganda stated that severe malaria was an important factor for high rates of malaria mortality and factors included mean duration of illness before getting antimalarial treatment, insecticide sprays, lack of protective measures, and season (11). The rainy season increased the risk of death in patients with severe malaria (18). Other factors associated with malaria include non-use of mosquito nets, respiratory diseases, seizures, hypoglycemia, incorrect drug administration, age, sex, delayed diagnosis, malaria involving *Plasmodium falciparum*, and poor immunity (20).

In Rwanda, malaria is a major public health burden that leads to mortality and morbidity among Rwandans, particularly children under five and pregnant women. In its improved health system, Rwanda has made efforts to fight against malaria through increasing awareness among the communities and all stakeholders in malaria responses and providing malaria prevention and control in all districts. Due to these efforts, there is a decrease in cases of severe malaria from 18,000 in 2016 to 3,000 in 2020. Based on previous Rwanda Demographic Health Surveys, Munini District Hospital in Nyaruguru district continues to report a higher rate of malaria cases and higher mortality rate due to severe malaria compared to the other districts of Rwanda. This communicable disease has led to high morbidity and mortality in Rwanda for several years (9). Studies show that malaria is a life-threatening disease that mostly occurs in young people and pregnant women in Rwanda. It was recently reported that the majority of cases were from the Eastern Province and Southern Province, which accounted for 80% of malaria cases in 2017 (21). To combat this infectious disease, the government of Rwanda has made efforts by providing mandatory health insurance for each Rwandan to cover treatment for malaria in all public health institutions. In addition, the government has implemented various strategies to prevent malaria, such as distributing insecticide-treated mosquito nets (ITNs) and undertaking indoor residual spraying (IRS) within households (13). Due to these preventive measures and access to medical treatments for malaria, the rate of morbidity and mortality have decreased (9).

The prevalence of malaria in the Nyaruguru district was 22% in 2016–2017 (HMIS) along with a high rate of mortality; however, factors associated with these high numbers remain unknown. Nyaruguru district, where the Munini hospital is located, is vulnerable to malaria as it is located in highlands and bordering endemic regions such as Huye district, Gisagara district, and Burundi. Therefore, the infection could come from outside. Despite the efforts made by the government and a significant reduction in malaria cases, the mortality rate remains high, particularly in the Southern and Eastern Provinces of Rwanda. Considering that malaria is the most prevalent killer disease among patients admitted to Munini hospital, we aimed to carry out this study to assess the malaria treatment outcomes and factors associated with malaria-related mortalities in this hospital. To the best of our knowledge, no other study has been carried out in Rwanda with this objective.

## Methods

### Study design and population

This study adopted a retrospective cross-sectional study design and was conducted in Munini District Hospital by extracting the data of patients from their hospital records. The study was restricted to patients diagnosed with severe malaria in Munini hospital, Nyaruguru district, Southern Province. The study population exhaustively included all patients admitted to Munini hospital due to severe malaria disease from 2016 to 2017. Since their total was 412, all of them were included in the study to maximize the study power. All medical records of admitted patients with severe malaria between 1 January 2016 and 31 December 2017 at Munini District Hospital were included in the study. Medical records with missing variables were excluded from the study. The inclusion criteria were: being a diagnosed adult (aged more than 18 years) and confirmed as a patient with severe malaria in the period between January 2016 and December 2017. Other inclusion criteria were: medical files with no missing data, medical files of persons with no other complicated diseases such as cancer, cardiovascular diseases, chronic pulmonary disease, and Type II Diabetes Mellitus. Additionally, this study included patients diagnosed by either quantitative buffy coat test or Leishman's stained peripheral blood smears with the presence of asexual forms of Plasmodium vivax or P. falciparum or both, with or without gametocytes. The study excluded medical files that had missing information, patients with non-communicable diseases and other complicated health conditions that could easily lead to mortality, patients with other health conditions including cognitive impairments, not providing written consent forms for participating, and having severe hearing and visual impairments. Medical files of patients who had coexistent non-malarial febrile illnesses were excluded. All patients were managed by the hospital clinicians in collaboration with the data collectors as per their clinical judgment and national guidelines.

### Study settings

**Figure 1:**
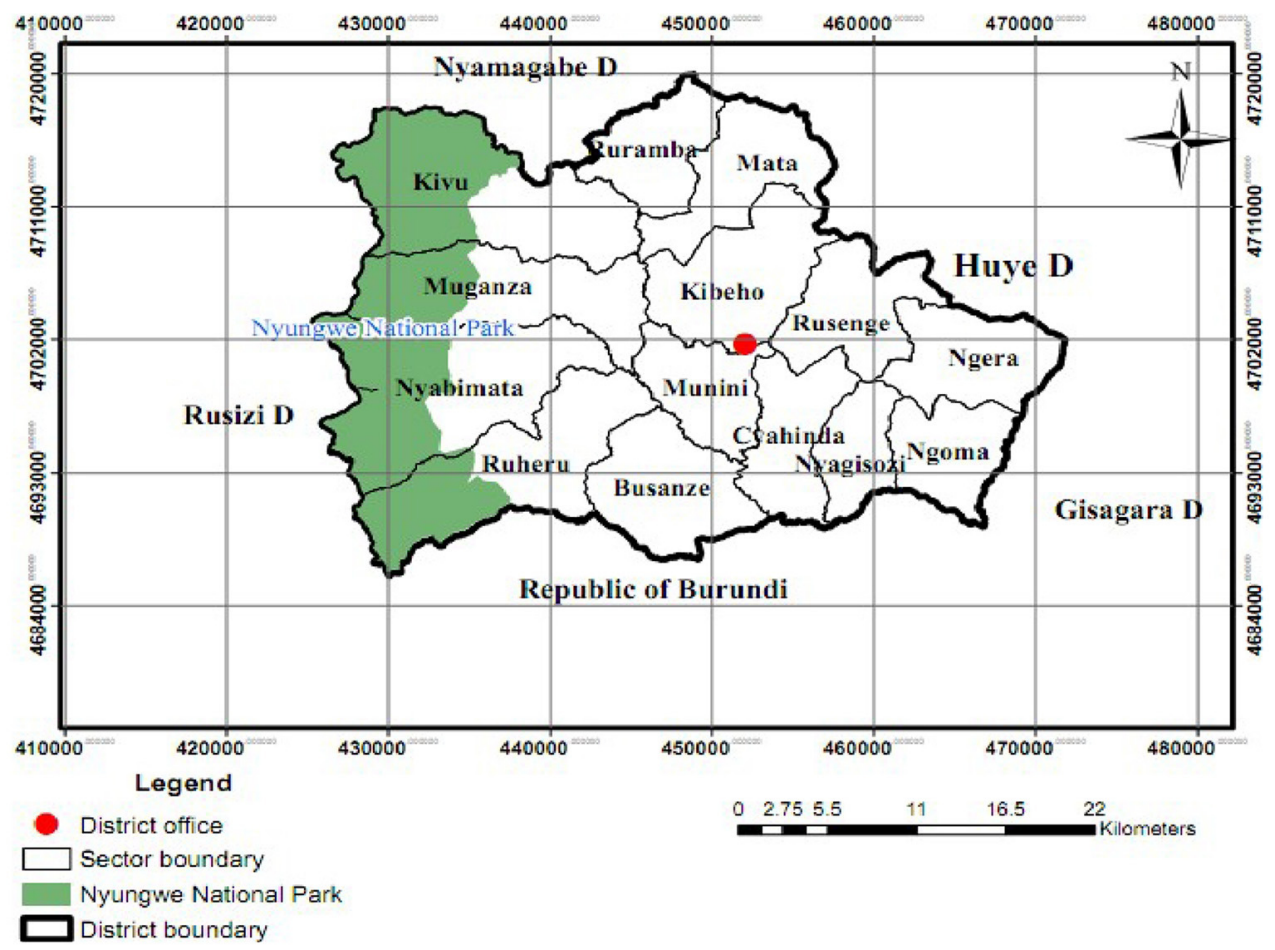
Administrative map of Nyaruguru District.

A retrospective cross-sectional study was conducted by reviewing the hospitalized records of malaria patients in Munini hospital, Nyaruguru district, Southern Province, Rwanda, from 2016 to 2017. Nyaruguru is one of the 30 districts in Rwanda and is located in its Southern Province. It is also one of the eight districts comprising the Southern Province. It is a mountainous district, containing part of the mountain forest of Nyungwe National Park and it is one of the popular tourist destinations. In the east, Nyaruguru district borders the Huye and Gisagara districts, and in the north, with Nyamagabe district. To its west, it shares its borders with the Western Province, and in the south, with the Republic of Burundi. Nyaruguru (Figure 1). has a surface area of 1,010 km^2^ comprising 14 sectors namely Busanze, Cyahinda, Kibeho, Kivu, Mata, Muganza, Munini, Ngera, Ngoma, Nyabimata, Nyagisozi, Ruheru, Ruramba, and Rusenge, which are made of 72 cells and 332 villages. The headquarter of this district is Kibeho, a pilgrimage site of the Catholics. Previous reports indicate that malaria is one of the communicable diseases that is a public health concern and the incidence of malaria in 2017–2018 was 230 per 1,000 people. The Munini District Hospital (22) is 164 km from Kigali city with a population of 323,624 habitants and is the only hospital in the whole district. The hospital has 72 beds with a fully functional minimum district package. All malaria cases admitted to Munini hospital from 2016 to 2017 were included in this study. Patients who did not fit within this period were excluded from the study. A data collection form that included all required variables was developed and used to collate data relating to malaria patients from the inpatient registers and files in compliance with data security and confidentiality.

### Study variables

The outcome variable was mortality. The independent variables included (a) socio-demographic characteristics such as age, sex, employment status, availability of health insurance, and proximity and access to health care; (b) clinical variables such as anemia, coma, abdominal pain, days with illness before consultation, disorientation, diarrhea, headache, joint pain, nausea, and vomiting; and (c) environmental variables such as season/weather.

### Data collection

Data collection was undertaken by data collectors whose background was in nursing. Medical records of patients with severe malaria were retrieved from the records rooms by taking all medical record numbers from the wards of adult patients who were admitted and discharged as per the registration log book. Before data collection, data collectors attended 2 days of training on the study objective. All data were collected from the medical files of patients admitted between 2016 and 2017. The medical records of the patients at the health facility were reviewed to assess the eligibility of the participants and then all relevant data were collected from those records.

### Statistical analysis

The collected data were entered into EpiData version 3.1. and then exported into STATA/MP Version.14.1 (Stata Corp 2015, College Station, TX, USA) for statistical analyses. Descriptive analyses were conducted to summarize the findings using tables using frequency and percentage. Univariate analysis was used for descriptive analysis. Then, in bivariate logistic regression analyses, significant variables at *p* < 0.05 were exported into multivariate logistic regression models to determine the association between independent and dependent variables. Then, the adjusted odds ratio was applied to determine associated factors of mortality due to severe malaria. Utilization of 95% for confidence intervals and statistical significance at *p* < 0.05 was ensured in the present study.

### Ethics

This study was reviewed and approved by the Institutional Review Board, College of Medicine and Health Sciences of the University of Rwanda with the reference number (CMHS/IRB/289/2019). It was also conducted in accordance with Helsinki Declaration and good clinical practices standards (22). The authorization to access the medical files of the patients was obtained from the directorate of the Ministry of Health through Munini District Hospital. No informed consent or assent was required to conduct the study because we used secondary data, but confidentiality was ensured. Data were unanimously collected.

## Result

The average age of the participants was 29.6 years (SD = 24), and the prevalence of mortality (treatment outcome) was 8% (*n* = 32). Most admitted patients were under 30 years old (62.84%). More women (59.1%) were admitted than men (41%). Approximately 78% of admitted patients were farmers and 93% had health insurance. The demographic details can be seen in Table 1.

**Table 1:** Description of the patients with severe malaria.

**Characteristics**	**Frequency**	**Percent**
**A. Socio demographic variables**		
**Age categories**		
0–4 years	39	9.73
4–15 years	106	26.43
16–30 years	107	26.68
31–45 years	51	12.72
46–60 years	36	9.98
61–101 years	62	15.46
**Proxy for access to healthcare**		
1 day	271	67.6
More than a day	130	32.4
**Sex**		
Men	164	40.9
Women	237	59.1
**Employment**		
Unemployed	27	6.73
Farmer	313	78.05
Student	54	13.47
Missing data	7	1.75
**Utilization of health insurance for seeking health service**		
Yes	373	93.02
No	28	6.98
**B. Clinical features**		
**Anemia**		
Yes	172	42.89
No	229	57.11
**Coma**		
Yes	111	27.6
No	290	72.4
**Abdominal pain**		
Yes	73	18.2
No	328	81.8
**Days with illness before consultation**		
1 day	271	67.6
More than a day	130	32.4
**Disorientation**		
Yes	132	32.9
No	269	67.1
**Diarrhea**		
Yes	25	6.3
No	376	93.7
**Fever**		
Yes	381	95
No	20	5
**Headache**		
Yes	139	34.66
No	262	65.34
**Joint pain**		
Yes	110	27.43
No	291	72.57
**Nausea**		
Yes	19	4.74
No	382	95.26
**Vomiting**		
Yes	348	86.78
No	53	13.22
**Prevalence of mortality**		
Yes	32	8
No	369	92
**C. Environment variables**		
**Season**		
Dry season	131	32.67
Rainy season	270	67.33

The univariate analysis showed that age was associated with malaria deaths (*p* < 0.001). Likewise, lack of health insurance was also associated with malaria death (*p* = 0.002). To be admitted being in a coma was associated with malaria death (*p* < 0.001). Similarly, mortality was associated with the duration of illness before consultation (*p* < 0.001). There was an association between malaria deaths and the season in which the patients were admitted (*p* < 0.01) (Table 2).

**Table 2:** Univariate analysis and number of deaths by socio-demographic and clinical characteristics.

	**Survive**		**Death**		
**Characteristics**	**Percent**	**Frequency**	**Percentage**	**Frequency**	***p*-Value**
**Age**					
0–4 years	37	10.03	2	6.25	
5–15 years	103	27.91	3	9.38	<0.001^***^
16–30 years	99	26.83	8	25	
31–45 years	49	13.28	2	6.25	
46–60 years	33	8.94	3	9.38	
61 years and above	48	13.01	14	43.75	
**Sex**					
Men	150	40.65	14	43.75	0.3
Women	219	59.35	18	56.25	
**Employment**					
No employment	26	7.05	1	3.13	
Farmer	287	77.78	26	81.25	
Student	49	13.28	5	15.63	0.6
Other	7	1.9	0	0	
**Utilization health insured**					
Yes	348	94.31	25	78.1	0.002^**^
No	21	5.69	7	21.9	
**Anemia**					
No	211	57	18	56.25	0.4
Yes	158	43	14	43.75	
**Coma**					
No	284	77	6	18.75	<0.001^***^
Yes	85	23	26	81.25	
**Abdominal pain**					
No	300	81.3	28	87.5	0.2
Yes	69	18.7	4	12.5	
**Proxy for access to healthcare**					
1 day	256	71.7	15	34.1	
More than a day	101	28.3	29	35.9	<0.001^***^
**Diarrhea**					
Yes	22	6	3	9	0.2
No	347	94	29	91	
**Fever**					
Yes	19	5.15	1	3.13	0.3
No	350	94.85	31	96.88	
**Headache**					
Yes	130	35	9	28.1	0.2
No	239	65	23	71.9	
**Joint pain**					
Yes	105	28	5	15.6	0.05
No	264	72	27	84.4	
**Nausea**					
Yes	17	4.6	2	6.3	0.3
No	352	95.4	30	93.7	
**Vomiting**					
Yes	49	13.3	4	12.5	0.4
No	320	86.7	28	87.5	
**Season**					
Rain	242	65.6	28	87.5	<0.001^**^
Dry	127	34.4	4	12.5	

* *: p < 0.05;*

** *: p < 0.01;*

*** *: p < 0.001*.

Our results from the bivariate logistic regression showed that age, coma, season, number of days with the disease without consultation, and having health insurance were significantly associated with death in patients with severe malaria. However, employment status, sex of patient, anemia, vomiting, fever, diarrhea, abdominal pain, joint pain, nausea, and headache were not significantly correlated with mortality among patients with severe malaria (Table 3).

**Table 3:** Bivariate logistic regression for analysis of the factors associated with death among the patients with severe malaria.

		**95% confidence intervals**		
**Variables**	**AOR**	**Lower limits**	**Upper limits**	***p*-Value**
**Age**				
0–4 years				0.002^*^
5–15 years	2.6	0.86	7.7	0.090
16–30 years	5.01	2.02	12.43	<0.001^***^
31–45 years	5	1.92	12.83	<0.001^***^
46–60 years	4.44	1.39	14.21	0.012^*^
61 years and above	4.16	1.13	15.36	0.033^*^
**Sex**				
Men	1			
Women	0.9	0.478	1.7	0.744
**Utilization of health insurance**				
No	1			
Yes	0.11	0.046	0.24	<0.001^***^
**Employment**				
No employment	1			0.886
Farmer	2.1	0.161	27	0.574
Student	1.37	0.16	11.7	0.775
Other	1.12	0.12	10.74	0.922
**Anemia**				
No	1			
Yes	1.246	0.665	2.333	0.493
**Coma**				
No	1			
Yes	6.48	3.32	12.68	<0.001^***^
**Abdominal pain**				
No	1			
Yes	0.55	0.21	1.43	0.219
**Proxy for access to healthcare**				
1 day	1			
More than a day	4.9	2.52	9.52	<0.001^***^
**Diarrhea**				
Yes	1			
No	1.6	0.52	4.9	0.410
**Fever**				
Yes	1			
No	1.12	0.25	5	0.887
**Headache**				
Yes	1			
No	1.21	0.64	2.31	0.558
**Joint pain**				
Yes	1			
No	0.47	0.2	1.08	0.075
**Nausea**				
Yes	1			
No	0.95	0.21	4.27	0.949
**Vomiting**				
Yes				
No	1.04	0.42	2.6	0.931
**Season**				
Rain	1			
Dry	0.39	0.15	1.02	0.032^*^

* *: p < 0.05;*

** *: p < 0.01;*

*** *: p < 0.001*.

Multivariate logistic regression models showed that malaria patients with coma were 7.32 times more likely to die (AOR = 7.32; 95%CI:3.33–16.1, *p* < 0.001) compared to those with no coma. Patients who had health insurance were less likely to die compared to those with no health insurance (AOR = 0.038; 95%CI:0.013–0.11, *p* < 0.001). Having severe malaria during the dry season reduced the risk of mortality (AOR = 0.23; 95%CI:0.08–0.64, *p* = 0.005). About age as a determining factor in severe malaria, our results showed that compared to patients aged 0–4, patients aged 16–30 had almost 6 times more risk of mortality (AOR = 1.91; 95%CI:1.91–17.5, *p* = 0.002). Similarly, 31–45 years olds were more at risk (AOR = 9.27, 95%CI:2.92–29.4, *p* < 0.001); so also patients over 61 years of age (AOR = 7.19, 95%CI:1.44–35.8, *p* = 0.016). The most vulnerable age group was the 46–60 group, which was 8 times more at risk (AOR = 7.78, 95%CI:2.06–29.34, *p* = 0.002). Patients who came for the consultation a day later had a greater likelihood to die (AOR = 3.7, 95%CI:1.8–4.1, *p* < 0.001) than those who came a day earlier (Table 4).

**Table 4:** Factors associated with death among the patients with severe.

**Variables**	**AOR**	**95%CI**	***p*-Value**
**Age**			
0–4 years	1		<0.001^***^
5–15 years	1.85	0.53–6.47	0.334
16–30 years	5.78	1.91–17.5	0.002^**^
31–45 years	9.27	2.92–29.4	<0.001^**^
46–60 years	7.78	2.06–29.34	0.002^*^
61 years and above	7.19	1.44–35.8	<0.016^**^
**Days with illnesses before consultation**			
1 day	1		<0.001^***^
More than a day	3.7	1.8–4.1	
**Coma**			
No	1		
Yes	7.19	3.33–16.1	<0.001^***^
**Utilization of health insurance**			
No	1		
Yes	0.038	0.01–0.11	<0.001^***^
**Season**			
Rain	1		
Dry	0.23	0.08–0.64	0.005^**^

* *: p < 0.05;*

** *: p < 0.01;*

*** *: p < 0.001*.

## Discussion

The purpose of our study was to investigate the factors associated with death among patients with severe malaria at the Munini District Hospital in the Southern Province of Rwanda from 2016 to 2017 which had a high prevalence of severe malaria. Our results revealed that 92% of all admitted malaria patients recovered while 8% died from malaria. The demography most affected by malaria infection were the young, represented by 9.73, 26.43, and 26.68% for age groups 0 to 4 years, 5–15years, and 16–30 years, respectively. These findings corroborate with findings from prior studies that indicated that the malaria infection rate varied from 8.2 to 34.3% among young adult students aged between 17 and 33 years of age (23, 24).

In accordance with previous studies (25), our results also revealed that most patients with severe malaria who were admitted and hospitalized were women. Our results are in line with a previous study that documented the prevalence of malaria and risk factors among patients attending Dutse General Hospital, Jigawa State, Nigeria where malaria prevalence was 52.8% in women and 48.9% in men. These increased numbers of severe malaria in women is because women often have lower immunity to malaria and lower hemoglobin level as well as an increased incidence of convulsion than their male counterparts. Women are also more likely to be exposed to malaria infections than men, especially due to their prolonged exposure to mosquito bites when mosquitos are most active. This finding was in line with previous studies (17, 30, 31).

Our results indicated that coma, age of the patient with severe malaria, dry season, and proximity to healthcare are important predictors of mortality among patients with severe malaria. Our results are supported by previous studies (26). The occurrence of severe malaria was 67.33% during the rainy season and 32.67% during the dry season. In our study, those who had severe malaria in the dry season were less likely to die when compared to those who had severe malaria in the rainy season. This reduction of mortality rate during the dry season is because malaria is more common in rainy seasons as most mosquitoes breed in waterlogged and damp places in the rainy season. In addition, in the dry season patients do not face any geographic barriers that may affect the visit to the Munini hospital which is at a high altitude. Our findings showed that the season is an important factor in malaria death; people who fell sick in the dry season were less likely to die when compared to those in the rainy season. Our results validate other studies conducted in sub-Saharan African countries (such as Malawi) that reported that the majority of deaths due to severe malaria occur in the rainy season (18). However, our results are divergent from other studies on seasonal associations and climatic drivers of malaria in the highlands of Ethiopia that reported that the incidence of malaria correlated with the dry season and it increased during this period rather than the rainy season (27).

To concur with previous studies conducted in developing countries (17–19), coma increased the risk of death due to severe malaria. Our results revealed that patients with severe malaria were 7 times more likely to die compared to patients who did not experience a coma. Our results revealed that the deaths due to severe malaria increased with age. For instance, those who were aged 16–30, 31–45, 46–60, and 61 years and above had greater likelihoods of mortality than those who were aged 0–4 years (28).

Patients who delayed coming to the hospital by a day were 4 times more likely to die than those who arrived a day earlier. This is in line with the finding that the longer a patient with severe malaria stays away without proper medical consultation by a professional healthcare provider the greater the patient's risk of mortality (29). The delay in the consultation was probably linked to the physical accessibility of the hospital and the community-based health insurance coverage in the whole Nyaruguru district. Besides, patients who had health insurance were less likely to die compared to others who did not. This is due to the delay in accessing health care when no health insurance is available. Most of the time patients with no health insurance get admitted when their condition has deteriorated and their chance of recovering is quite slim.

To the best of our knowledge, this is the first exclusive study describing the outcomes of malaria in adults. Sufficiently large cohort and robust statistical analysis provide significant strength to the study findings. Furthermore, stratified evaluation of factors for their association with outcomes across malaria cohort, severe and non-severe subgroups confers additional visibility.

Our study had some limitations. This was a retrospective cross-sectional study using secondary data analysis of malaria treatment outcomes among patients admitted to the hospital from 2016 to 2017. So, this study design was unable to determine the causality of the malaria outcome in adults. Further, this was a hospital-based study that did not permit us to generalize based on the present results; so, information on concurrent non-febrile comorbidities was not collected for this research and as a result, their effects on malaria outcome could not be deduced. Another limitation was that some important variables were not collected because they were not in the patient registries or medical files such as the incidence of previous disease or comorbidity, respiratory distress, delayed diagnosis and treatments, hypoglycemia and acidosis, impaired consciousness, HIV status, comorbidities, respiratory pain, hypertension, jaundice, mean duration of illness before getting antimalarial drugs, insecticide sprays, lack of protective measures, seizures, incorrect drugs administration, shock, bleeding, pulmonary edema, renal impairment, prostration, extreme weakness, urinary tract infection, kidney enteritis, retroviral infection, malaria involving Plasmodium falciparum, and immunity of the patient. Further investigation into socio-biophysical factors of the deaths among patients with severe malaria is recommended. Future scholars are recommended to conduct studies on malarial outcomes involving ample subjects each with and without the study outcomes to determine more vigorous association. Prominently, a multicentric research method among patients with malaria would be significantly important to evaluate and determine the extent of subjectivity associated with criteria for hospitalization and intensive unit care across hospitals within different regions.

## Conclusion

In conclusion, our study demonstrated that malaria deaths among patients admitted to Munini hospital from 2016 to 2017 were highly associated with a lack of health insurance, a delay in consultation, and seasonal factors as well as coma complicated malaria, as discussed above. Efforts must be put in for community sensitization, early consultation of malaria patients, and availing health insurance each year to overcome the problem of delay in consultation. However, hospital health care professionals also have to improve their efforts in severe malaria cerebral form management as well as other severe forms of malaria, given that they are the most likely to be admitted at the district level. Designing preventive measures and providing continuous training to nurses on severe malaria case management, mainly cerebral malaria, are recommended. Policymakers are recommended to improve the effectiveness of community health workers in malaria early consultation, diagnosis, and management and make mosquito nets available to all households very early.

## Data availability statement

The raw data supporting the conclusions of this article will be made available by the authors, without undue reservation.

## Ethics statement

The studies involving human participants were reviewed and approved by College of Medicine and Health Sciences, University of Rwanda. The patients/participants provided their written informed consent to participate in this study.

## Author contributions

FH, JO, and JN conceptualized the study and designed the methods. JN supervised the study and provided substantial contributions to the interpretation of data and took part in revising the paper critically for important intellectual contents. EB contributed to the data analysis and drafted the manuscript. FH collected data and analyzed the data. All authors agreed to submit to the current journal, accountable for all aspects of the work, and provided final approval of the version to be published.

## Conflict of interest

The authors declare that the research was conducted in the absence of any commercial or financial relationships that could be construed as a potential conflict of interest.

## Publisher's note

All claims expressed in this article are solely those of the authors and do not necessarily represent those of their affiliated organizations, or those of the publisher, the editors and the reviewers. Any product that may be evaluated in this article, or claim that may be made by its manufacturer, is not guaranteed or endorsed by the publisher.
